# Progression of COVID-19 in a Patient on Anti-CD20 Antibody Treatment: Case Report and Literature Review

**DOI:** 10.1155/2022/8712424

**Published:** 2022-02-25

**Authors:** Sebastian Burgener, Philippe Rochat, Günter Dollenmaier, Gabriel Benz, Andreas D. Kistler, Rosamaria Fulchini

**Affiliations:** ^1^Department of Internal Medicine, Cantonal Hospital Frauenfeld, Frauenfeld, Switzerland; ^2^Center for Laboratory Medicine, St. Gallen, Switzerland; ^3^Center for Sleep Medicine, Department of Pneumology and Sleep Medicine, Cantonal Hospital St. Gallen, St. Gallen, Switzerland; ^4^Department of Internal Medicine, Infectious Diseases and Hospital Epidemiology, Cantonal Hospital Münsterlingen, Münsterlingen, Switzerland

## Abstract

Accumulating evidence suggests that anti-CD20 treatments are associated with a more severe course of COVID-19. We present the case of a 72-year-old woman treated with the B-cell-depleting anti-CD20 antibody rituximab for seropositive rheumatoid arthritis with severe acute respiratory syndrome coronavirus 2 (SARS-CoV-2) infection causing a clinical relapse more than 4 weeks after the first manifestation. Persistently positive reverse transcription polymerase chain reaction (RT-PCR) results along with a drop in cycling threshold (Ct) values, in addition to recovery of identical viral genotype by whole genome sequencing (WGS) during the disease course, argued against reinfection. No seroconversion was noted, as expected on anti-CD20 treatment. Several other case reports have highlighted potentially fatal courses of COVID-19 associated with B-cell-depleting treatments.

## 1. Introduction

Immunocompromised patients are at risk for severe disease courses of COVID-19 and prolonged viral shedding [1]. At the beginning of the pandemic, little was known about COVID-19 patients on anti-CD20 antibody treatment like rituximab, causing prolonged B-cell depletion. However, there is now growing evidence that rituximab is associated with adverse outcomes of COVID-19 infection [2]. Here, we report the intriguing case of a 72-year-old woman receiving rituximab for seropositive rheumatoid arthritis, who experienced a relapsing course of COVID-19. We performed a literature search for case descriptions, including patients with COVID-19 on anti-CD20 antibody treatment.

## 2. Case Presentation

A 72-year-old woman was tested positive for SARS-CoV-2 by RT-PCR in a nasopharyngeal swab on the 4^th^ of February 2021. Her past medical history was remarkable for seropositive rheumatoid arthritis and connective tissue disease-associated interstitial lung disease, for which she received rituximab treatment biannually. Five months before COVID-19 onset, she had received her last dose. Other comorbidities included pulmonary arterial hypertension, coronary artery disease, and atrial fibrillation. At the early stage of the national vaccination campaign, our patient had not been vaccinated. Twelve days later, she was hospitalized in another hospital due to respiratory failure. In addition to the known interstitial lung disease, chest computed tomography revealed new bilateral ground glass opacities consistent with COVID-19 infiltrates. The patient received supplemental oxygen, dexamethasone, and a 10-day course of remdesivir. Following her gradual improvement, a nasopharyngeal SARS-CoV-2 antigen test turned out negative on day 22 and follow-up Ct values of RT-PCR were increasing; thus, isolation precautions were stopped. On day 27, the patient was discharged to inpatient pulmonary rehabilitation. Thirty-three days after diagnosis, she was admitted to our hospital because of relapsing dyspnea, productive cough, and fever, along with respiratory failure requiring nasal high-flow oxygen therapy and intensive care monitoring. Differential diagnoses included bacterial pneumonia, reinfection with a SARS-CoV-2 mutant, or a relapse of preexisting COVID-19. Inflammatory markers were again elevated, and a computed tomography scan was unchanged (Table 1).

We started treatment with broad-spectrum antibiotics, but sputum analysis and Legionella antigen in the urine were negative. Further invasive measures like mechanical ventilation were not pursued, according to the patient's wishes. 35 days after the first positive RT-PCR test, the patient died from worsening respiratory failure. Autopsy revealed acute diffuse alveolar damage with hyaline membranes due to COVID-19 in addition to underlying interstitial lung fibrosis.

**Figure 1:**
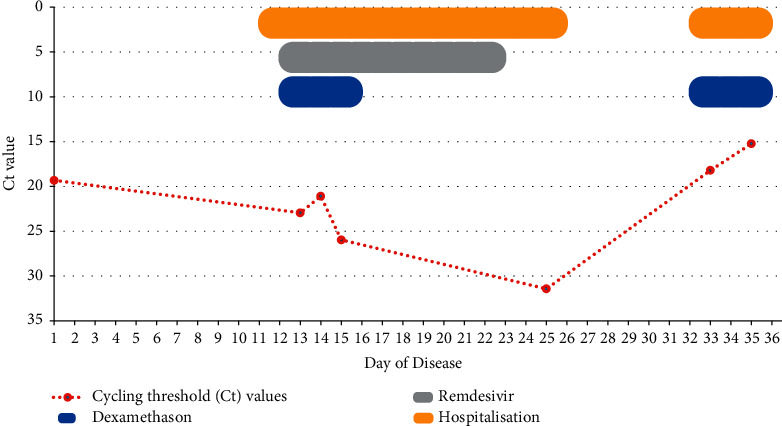
Timeline of cycling threshold (Ct) values during disease course.

Follow-up of Ct values of RT-PCR during the second course of disease showed a clear drop, which was indicative of a recurrence of previous COVID-19 (Figure 1).

To rule out reinfection by a mutant, we performed whole genome sequencing of SARS-CoV-2 of the first and last nasopharyngeal swab isolates (days 0 and 35, respectively). Viral genotypes showed identical sequence patterns, and mutational analysis for N501Y and E484K was negative. Furthermore, SARS-CoV-2 serology was not able to detect IgG or IgM antibodies. These findings led to the conclusion that our patients' clinical deterioration was due to a prolonged and relapsing course of COVID-19.

## 3. Discussion

Our literature review showed accumulating evidence for variable clinical courses of COVID-19 in patients with functional B-cell immunodeficiency due to treatment with rituximab (Table 2).

Reported patients mostly suffered from hemato-oncological or rheumatological comorbidities as an indication for rituximab treatment. Prolonged shedding of SARS-CoV-2 in nasopharyngeal swabs along with either a benign or adverse outcome is a notable feature, independent of the underlying disease. To our knowledge, only one case report has provided whole genome sequencing to rule out reinfection during a prolonged clinical course [2]. This was an important differential diagnosis in our case, since our patient's clinical worsening occurred during the epidemiological situation of a nationwide third wave of COVID-19 with upcoming variants of concern. The substantial drop in Ct values during the second course of disease could not be explained by preanalytical sampling differences [18], and reinfection was ruled out by genetically identical viral variants of the first and last isolate. A possible hypothesis explaining the initial decrease in viral load, based on in vitro data but not confirmed by clinical studies [19, 20], could be that remdesivir reduced viral replication, but after stopping antiviral treatment, viral clearing was not possible due to an insufficient antibody response and re-emerging viral replication led to a clinical relapse. The potentially severe COVID-19 course under rituximab suggests that, in addition to cellular immunity, humoral immunity plays an important role. Therefore, monitoring of immunocompromised patients with B-cell depletion after stopping antiviral treatment is crucial, and repeat quantitative RT-PCR, in addition to clinical assessment, might be useful to detect re-emerging viral replication and infectivity.

Our single case description may not be generalizable to other immunocompromised populations. However, cases with relapsing and prolonged courses have been attributed to reduced viral clearance due to the lack of anti-SARS-CoV-2 antibody production by prolonged B-cell depletion after anti-CD20 therapy, as was the case in our patient [3, 5, 7, 10, 17].

This case also highlights the infection control challenges in the handling of this special population with persistent shedding of potentially viable virus.

In conclusion, caution should be taken in patients with anti-CD20 antibody treatment, as they can acquire SARS-CoV-2 infection despite vaccination because of the lack of antibody emergence and prolonged or relapsing disease courses have to be expected.

## Figures and Tables

**Table 1:** Timeline of clinical and laboratory data during hospitalisation.

Days after symptom onset	Day of admission to the other hospital, day 12	Day of discharge from the other hospital, day 25	Day of admission to our hospital, day 33	Day of death, day 35
SARS-CoV-2 PCR (nasopharyngeal swab)	Positive	Positive	Positive	Positive
Ct value	23.0	31.4	18.2	15.2
Heart rate (beats/min)	95	65	126	135
Blood pressure (mmHg)	137/87	183/89	124/80	179/83
Fever (°C)	36.6	36.4	38.1	38.2
Breathing rate (breaths/min)	20	23	25	32
SpO_2_ (%)	99	94	100	89
FiO_2_ (%)	25	21	80	80
White blood cell count (x10^9^/l)	5.5	19.2	17.2	14.4
C-reactive protein (mg/l)	152	5	220	224
Ferritin (mcg/l)				3446
D-dimer (mg/l)				3.22
Lactate dehydrogenase (U/I)	550			793

**Table 2:** Literature search of patients with COVID-19 and receiving immunosuppressive treatment including rituximab.

Study	Number of patients	Age (years)	Gender	Underlying disease	Repetitive positive respiratory sample by RT-PCR^a^	Duration of symptoms (days)	Cumulative hospital-days (days)	Outcome
Avouac et al. [2]	63	59^d^	25 males	Inflammatory rheumatic and musculoskeletal diseases	NA	NA	13^d^	13/63 died
			38 females					

Baang et al. [3]	1	60	Male	Mantle cell lymphoma	Yes	NA	6	Survived

Sepulcri et al. [4]	1	60–70	Male	Mantle cell lymphoma	Yes	271	268	Died

Lancman et al. [5]	1	55	Female	B-cell lymphoma	Yes	55^c^	40^c^	Survived

Choi et al. [6]	1	45	Male	Antiphospholipid antibody syndrome	Yes	NA	5	Died

Friedman and Winthrop [7]	1	30	Female	Granulomatosis with polyangiitis	Yes	Several weeks	NA	Survived

Leipe et al. [8]	1	63	Male	Granulomatosis with polyangiitis	NA^b^	32	30	Survived

Tepasse et al. [9]	2	65	Male	Cerebral diffuse large B-cell lymphoma	NA^b^	23	22	Died

		66	Male	Mantle cell lymphoma	NA^b^	30	26	Died

Benucci et al. [10]	1	60	Female	Polymyositis and Sjögren syndrome	Yes	63	63	Survived

Yasuda et al. [11]	1	61	Female	Follicular lymphoma	Yes	59^c^	59	Survived

Guilpain et al. [12]	1	52	Female	Granulomatosis with polyangiitis	Yes	29	25	Survived

Schulze-Koops et al. [13]	2	71	Male	Rheumatoid arthritis	No	14	12	Died
		80	Female	Rheumatoid arthritis	No	17	17	Died

Kos et al. [14]	1	72	Male	Nodal marginal zone lymphoma	Yes	31^c^	24	Survived

Fallet et al. [15]	1	77	Female	Granulomatosis with polyangiitis and Sjögren syndrome	No	6^c^	6	Survived

Wurm et al. [16]	1	59	Female	Multiple sclerosis	Yes	15	13	Survived

Pascale Daniel et al. [17]	1	55	Male	Granulomatosis with polyangiitis	Yes	29	22	Survived

^a^Tests drawn by nasopharyngeal swab, sputum, or bronchoalveolar lavage were considered. ^b^The method of testing is not described. ^c^Approximate values have been applied, because exact numbers were not documented. ^d^Mean values.

## Data Availability

The data supporting the results are available, on request, from the authors.
